# Emerging roles of low-density lipoprotein in the development and treatment of breast cancer

**DOI:** 10.1186/s12944-019-1075-7

**Published:** 2019-06-10

**Authors:** Xuefeng Guan, Zhuo Liu, Zhen Zhao, Xuefeng Zhang, Siteng Tao, Bao Yuan, Jiabao Zhang, Dawei Wang, Qing Liu, Yu Ding

**Affiliations:** 1 Department of Laboratory Animals, College of Animal Sciences, JiLin University in Changchun of Jilin Province in China, Xian Road 5333#, Changchun, 130062 China; 2 Guangdong Provincial Hospital of Chinese Medicine-Zhuhai Hospital, Zhuhai, 519015 China; 3 China-Japan Union Hospital of Jilin University, Changchun, 130333 China; 4 College of Animal Science and Technology, Jilin Agricultural University, Changchun, 130118 China; 5 Department of Immunology, School of Basic Medical Sciences, Fudan University, Shanghai, 200032 China; 6 The 2nd Clinical School of Medicine, Guangdong Provincial Hospital of Chinese Medicine, Guangzhou University of Chinese Medicine, Guangzhou, 510120 China; 7 The 85th Hospital of CPLA, Shanghai, 200040 China

**Keywords:** Breast cancer, LDL, Heterogeneous, Progression, Prognosis, Treatment

## Abstract

Breast cancer is a heterogeneous disease with increasing incidence and mortality and represents one of the most common cancer types worldwide. Low-density lipoprotein (LDL) is a complex particle composed of several proteins and lipids, which carries cholesterol into peripheral tissues and also affects the metabolism of fatty acids. Recent reports have indicated an emerging role of LDL in breast cancer, affecting cell proliferation and migration, thereby facilitating disease progression. However, controversy still exists among distinct types of breast cancer that can be affected by LDL. Classical therapeutic approaches, such as radiotherapy, chemotherapy, and lipid-lowering drugs were also reported as affecting LDL metabolism and content in breast cancer patients. Therefore, in this review we summarized and discussed the role of LDL in the development and treatment of breast cancer.

## Introduction

Breast cancer is one of the most commonly diagnosed type of cancer, ranking the second-leading cause of cancer-related deaths in women [1]. The pathological role of lipids was indicated when a relationship between obesity and breast cancer was observed [2]. Subsequent studies have shown that obesity may be the cause of a large percentage of breast cancer incidence [3–5] and mortality, implying that abnormal or excessive lipid accumulation may affect breast cancer progression, prognosis, and treatment. Therefore, in this review we summarized and discussed the role of lipids in breast cancer.

### Metabolism of low-density lipoprotein-cholesterol

LDL is a cholesterol-rich lipoprotein particle that carries cholesterol into peripheral tissues, and can be oxidized to form oxidized LDL. At present, two sources of LDL in the plasma are known: the main source derives from VLDL metabolism, and the second source derives from liver synthesis and subsequent secretion into the circulation [6–8].

LDL is internalized into the cell through receptor-mediated endocytosis [9]. The covered lacuna on the cell membrane surface is the site of LDL receptor. Once the LDL is bound to its receptor, an endocytic vesicle is formed. Membrane-associated vacuolar H + -ATPase maintains an acidic pH in the endocytic vesicle by pumping cytosolic H+ into the lumen in an ATP-dependent manner. Once the vesicle is internalized, the receptors return to the plasma membrane, while LDL is delivered to the lysosomes where cholesterol is released, and either exits the lysosomes or is re-esterified by ACAT for accumulation An LDL percentage of 65–70% in blood plasma depends on LDL receptor clearance, and a small fraction (about 1/3) is incorporated by surrounding tissues (including vessel walls). In the event of LDL deficiency, the remaining VLDL is recognized by most LDL receptors, of which most is converted to LDL, thereby increasing the plasma LDL concentration [10–12].

**Fig. 1:**
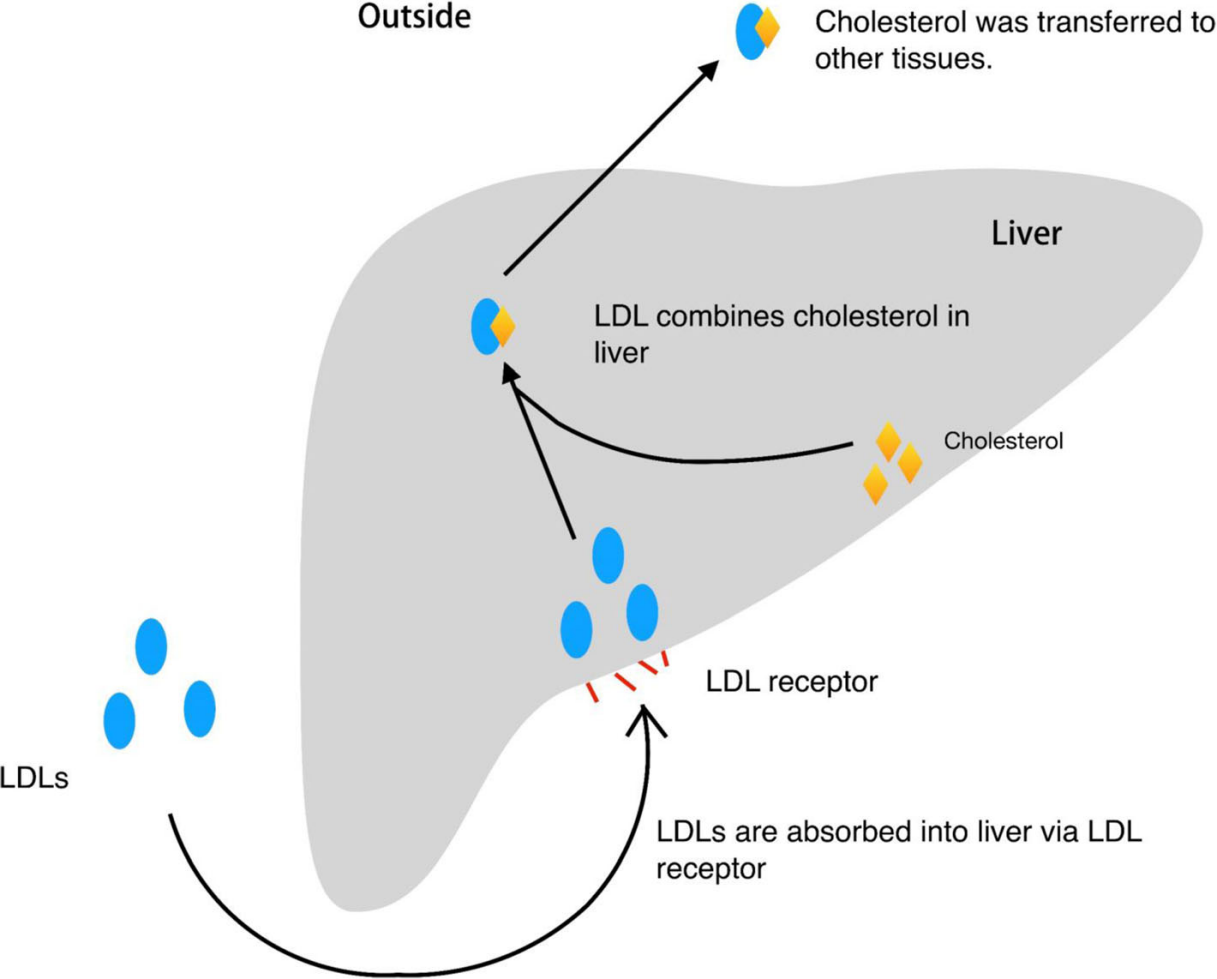
LDLs are combined with LDL receptors and absorbed into liver, then LDL transferred cholesterols by combination

Cholesterol is an oil-oil complex that is predominantly produced by the liver. The total amount of cholesterol in the human body is 100 to 200 g. Two-thirds of this is derived from self-synthesis in the body, whereas one-third is derived from food. Cholesterol must be combined with lipoproteins to be transported to various parts in the body. Lipoproteins include LDL and high-density lipoproteins (HDL) [13]. LDL transports cholesterol from the liver to other organs, whereas HDL transports cholesterol from the other organs back to the liver where it is metabolized [14–16]. The metabolic process above is shown in Fig. 1.

### LDL increases breast cancer progression

LDL affects the adhesion of breast cancer cells, favoring cell migration and proliferation. Catarina et al. investigated the function of LDL in MDA-MB-231 breast cancer cells by treating the cells with LDL (100 μg/mL) for 24–48 h, and discovered that breast cancer cell proliferation was increased. Furthermore, LDL induced MDA-MB-231 cell migration and decreased adhesion [10, 17, 18]. When comparing the expression of different genes in LDL-treated and control cells at 48 h, overexpression (fold change ≥1.5) of 147 mapped genes (including pERK, pAKT, and pJNK) was observed, whereas 95 mapped genes were down regulated (including CD226, Claudin7, Ocludin, and integrinβ8) [19, 20]. The majority of these genes were involved in pathways regulating cell survival and proliferation. In a subsequent study they confirmed increased phosphorylation of Akt and ERK, but not JNK [10]. While NMT1 inhibition modulates breast cancer progression through stress-triggered JNK pathway [21, 22]. Furthermore, their results indicated that some genes changed their expression without modifying the protein level, whereas for other genes, both the gene expression and corresponding protein expression was changed. Next, they fed mice a high cholesterol diet, which resulted in high LDL levels, and subsequently MDA-MB-231 and 4 T1 cells (another breast cancer cell line) were inoculated in mice, revealing that these cells in high cholesterol diet-fed mice showed a higher proliferative ratio when compared to that of cells inoculated in mice that were fed a normal diet [23–26].

### LDL functions as a prognostic indicator for breast cancer

Vipan et al. evaluated the role of serum lipid content in 100 breast cancer patients and healthy women. Their results revealed that patients with breast cancer possessed high levels of LDL, triglycerides, and total cholesterol when compared to healthy women. No differences were observed in VLDL and HDL levels between patients and healthy women [27]. Catarina et al. evaluated whether plasma LDL-cholesterol levels could be a predicting factor for breast cancer by enrolling 244 breast cancer patients. The results demonstrated that the LDL-cholesterol fraction was significantly associated with breast cancer progression, which may be critical in identification and follow-up of high-risk breast cancer patients. Therefore, LDL-cholesterol levels at the time of diagnosis may be considered as a prognostic factor in breast cancer [28].

Besides LDL, Xing et al. studied the effect of preoperative serum triglycerides and HDL-cholesterol levels in the prognosis of breast cancer. Their results showed that a preoperative lower HDL-cholesterol level was a risk factor in breast cancer patients, and decreased levels were significantly associated with worse overall survival [29, 30]. Paulette et al. evaluated the association between lipid biomarkers and long-term cancer risk, and their on-patients investigation revealed that total cancer risk was significantly lower in the highest quartile of apo A-I and HDL-cholesterol [31].

### LDL functions differently in distinct breast cancer types

Lu et al. divided LDL into five charge-based subfractions, L1-L5, using anion exchange chromatography, and investigated the relationship between angiogenesis and these subfractions. After treating MCF-7 (luminal-like) cells and MDA-MB-231 (basal like) cells with L1 and L5, they found that L1 and L5 did not effect cell viability of MCF-7 cells, but enhanced the viability of MDA-MB-231 cells. Furthermore, they proved that L1 and L5 enhanced anchorage-independent growth, a hallmark of oncogenic transformation, in MDA-MB-231 cells. They also found that L1 had no significant effect on cell migration in MCF-7 cells but increased the migration of MDA-MB-231 cells. In addition, L1 and L5 increased cell migration in other two basal-like breast cancer cell lines, MDA-MB-468 and HS578T. They further discovered that L1 and L5 enhanced the phosphorylation of Akt at S473 in MDA-MB-231 cells, and the phosphorylation of Stat3 at Y705, but not the phosphorylation of Erk, p38, or NF-kB [32].

**Fig. 2:**
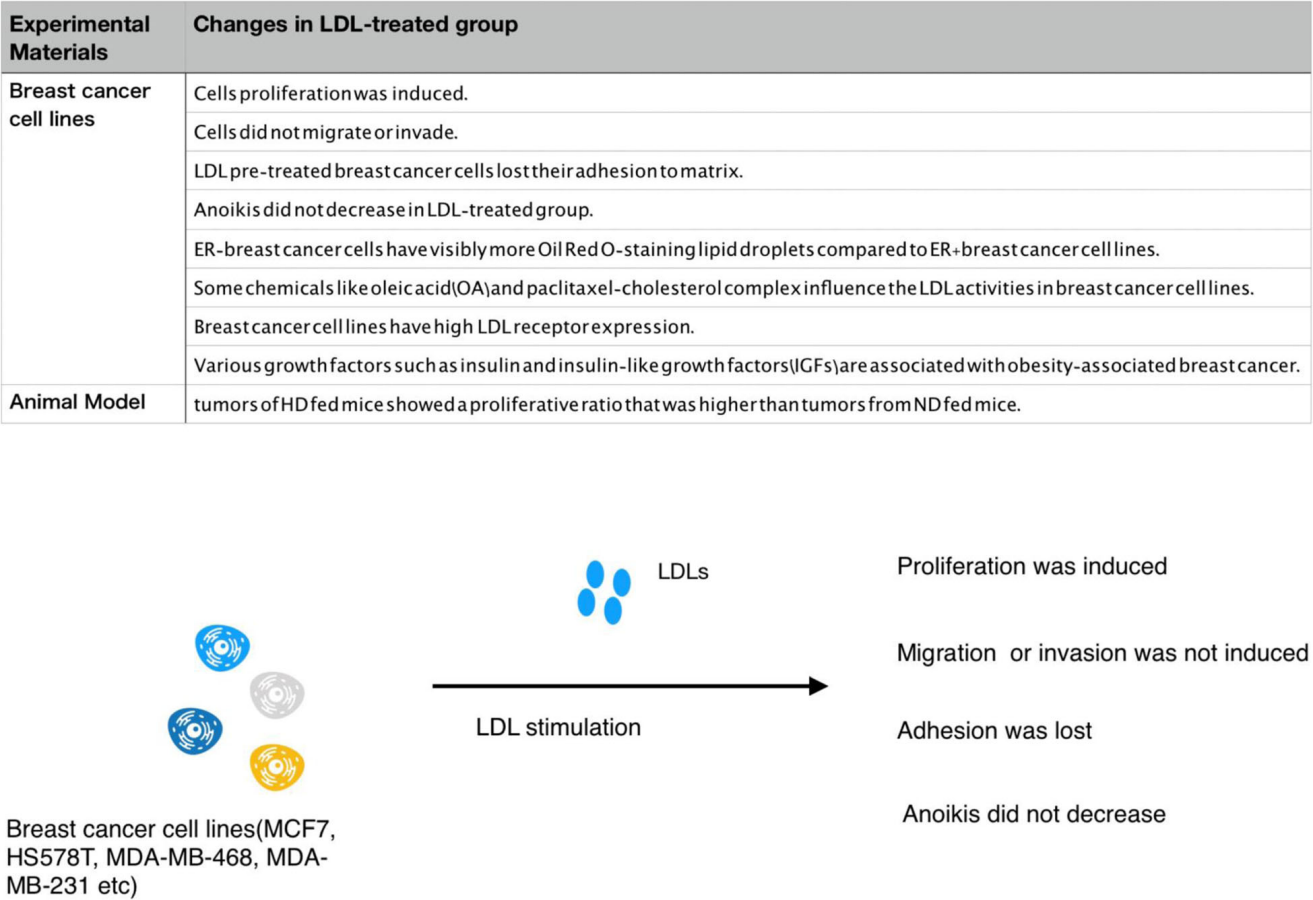
We conclude the function that LDL matters to breast cancer cel lines like MDA-MB-231 cells and HTB-20 cells

Caryl et al. investigated the uptake of LDL by breast cancer cells by evaluating LDL receptor expression. They treated MDA-MB-231, MDA-MB-436, MCF-7, and MCF-10A cells with LDL and stained the cells with Oil Red O [33–35]. Their findings revealed that under identical growth conditions, ER-cells, such as MDA-MB-231 and MDA-MB-436 showed elevated Oil Red O-stained lipid droplets when compared to ER+ cell, such as MCF-7. Basal and stimulated neutral lipid formation was measured after oleic acid (OA) treatment for 6 h. The results showed that OA treatment increased lipid droplet formation in all cell types. Baseline total triacylglycerol concentration was lower in ER+ MCF-7 cells when compared to ER- MDA-MB-231 and MDA-MB-436 cells, and OA treatment stimulated total triacylglycerol accumulation to a lesser extent in MCF-7 cells. MCF-7 cells also showed a lower baseline concentration of CE that did not increase in response to OA. In contrast, ER- MDA-MB-231 and MDA-MB-436 cells had a higher baseline CE concentration and showed a dose-dependent increase in CE in response to OA. These results demonstrated a difference in both quantity and type of neutral lipid synthesis and storage in basal-like ER- breast cancer cells compared to luminal ER+ cells [36, 37]. Functions on animal models caused by LDL are summarized in Fig. 2.

### LDL influences the effects of fatty acids on breast cancer

Long-chain polyunsaturated fatty acids (PUFAs) such as omega-3 (n-3) are thought to have tumor-suppressing properties. Specifically, n-3 PUFAs promote the apoptosis of breast cancer cells [38]. and n-3 PUFA-enriched low-density lipoprotein (LDL) has been found to increase the production of syndecan-1 in breast cancer cells and inhibit their development [39]. Moreover, omega-6 (n-6) PUFAs can promote the proliferation and migration of breast cancer through phospholipase D (PLD) and free fatty acid receptor 1 (FFAR1) [40]. It has been found that in MCF-7 and MDA-MB human breast cancer cells, n-3 PUFAs can effectively promote the expression of breast cancer suppressor genes BRCA1 and BRCA2. Interestingly, although n-6 PUFAs have been found to induce tumor proliferation, no effect on the expression of BRCA1 and BRCA2 was found in cells treated with them [41, 42]. Concurrently, it has been pointed out that n-3 PUFAs had a dose-dependent effect on the fatty acid composition of breast adipose tissue in women with a high risk of breast cancer in a 6-month randomized open-label study involving 48 women with an increased risk of breast cancer [43, 44]. Overall, n-3 PUFAs inhibit breast cancer and n-6 PUFAs promote breast cancer.

N-3 and n-6 PUFAs enter organisms mainly through dietary intake and supplements and are then transported to the tissues by cholesterol, very LDL (VLDL), or LDL, or delivered to the liver via high-density lipoprotein (HDL) [13, 14]. Compared with albumin, more n-3 PUFAs bind to tumor receptors through LDL, thereby inhibiting tumor growth and promoting apoptosis.

In general, LDL has a high concentration of fatty acids [45] However, an excessive intake of n-6 PUFAs can lead to a reduction in LDL receptors, which disables n-3 PUFA and promotes the migration and proliferation of tumors. Unfortunately, an improvement in n-3 PUFA intake by healthy subjects does not alter the known effects of dietary saturated fats or n-6 PUFAs on LDL [46] N-3 PUFA consumption reduces the liver’s secretion of triglyceride-rich lipoprotein (TRL) and enhances its conversion to LDL. Moreover, they also reduce the concentration of apolipoprotein B (Apo B)-48 TRL by reducing its secretion. It has also been found that high n-6 PUFA intake reduces VLDL cholesterol and triglyceride concentrations by the upregulation of lipolysis and the uptake of VLDL [47].

### Radiotherapy affects lipid metabolism in breast cancer

Radiotherapy is a classical therapeutic approach for the treatment of breast cancer. Sana et al. studied how radiotherapy affected lipids metabolism, including serum LDL levels and total cholesterol in breast cancer patients. Their results showed that serum total cholesterol and LDL-cholesterol are significantly decreased after the radiotherapy treatment [48]. However, in another study by Ozmen et al., it was revealed that HDL-cholesterol and total cholesterol levels decreased, while triglycerides and LDL-cholesterol levels increased in patients subjected to radiotherapy compared to healthy controls [49]. This difference may be due to the heterogeneity of the selected subjects in each clinical trial, thus, more investigations should be performed in order to reveal the underlying mechanism involved in the change of lipids during radiotherapy.

Interestingly, not only radiotherapy affects LDL levels, but lipids including LDL also affect the sensitivity of breast cancer cells to radiotherapy. Wolfe et al. found that HDL and LDL have opposite roles compared to VLDL in regulating tumor-initiating cells and sensitivity to radiation in inflammatory breast cancer. VLDL increases and HDL decreases mammosphere formation when compared to untreated SUM 149 and KPL4 cells. In inflammatory breast cancer patients, higher VLDL levels (> 30 mg/dL) predicted a lower 5-year overall survival rate, while lower HDL levels (< 60 mg/dL) predicted a lower 5-year overall survival rate [50].Although further studies are needed to confirm and interpret these observations, this study at least discovered a relationship among plasma lipoproteins levels, overall patient response, and radiation resistance in inflammatory breast cancer patients.

### Neoadjuvant chemotherapy affects lipid metabolism in breast cancer

Besides radiotherapy, the effect of neoadjuvant chemotherapy on LDL receptors and LDL receptor-related protein 1 receptor was investigated in patients with locally advanced breast cancer. Changes in LDL receptor expression did not reach a statistical significance due to the small amount of subjects [51]. Both Che et al. [52] and Love et al. [53] studied the influence of tamoxifen in the lipid profile of breast cancer patients, and their results revealed that tamoxifen significantly lowers the serum lipid profile of breast cancer patients, including serum LDL-cholesterol and total cholesterol. These results are also supported by a study performed byDziewulska et al [54] Consistently, Chow et al. investigated postmenopausal women with breast cancer, showing that LDL-cholesterol levels in patients treated with exemestane 25 mg daily and celecoxib 400 mg twice-daily progressively decreased, and a statistical significance was observed between the fifth week after operation and preoperative levels [55].

Results from the study by Wasan et al. suggested that letrozole does not significantly alter serum cholesterol, HDL-cholesterol, LDL-cholesterol, triglycerides or Lp(a) in non-hyperlipidemic postmenopausal women with primary breast cancer, although treated with it up to 36 months following at least 5 years of adjuvant tamoxifen therapy [56]. Moreover, Lu et al. showed that anastrozole may lead to an abnormal lipid metabolism in postmenopausal patients with breast cancer. Anastrozole significantly increased the levels of LDL-cholesterol, total cholesterol and HDL-cholesterol, and significantly reduced the level of triglycerides [57]. These results suggested that blood lipid levels should be regularly assessed in breast cancer patients under long-term anastrozole treatment, while this assessment seems not relevant in patients under letrozole treatment. The studies mentioned above also suggested that baseline lipid levels in breast cancer patients may affect the effect of neoadjuvant chemotherapy on the lipid profile in breast cancer patients.

### Lipid-lowering drugs affect lipid metabolism in breast cancer

**Fig. 3:**
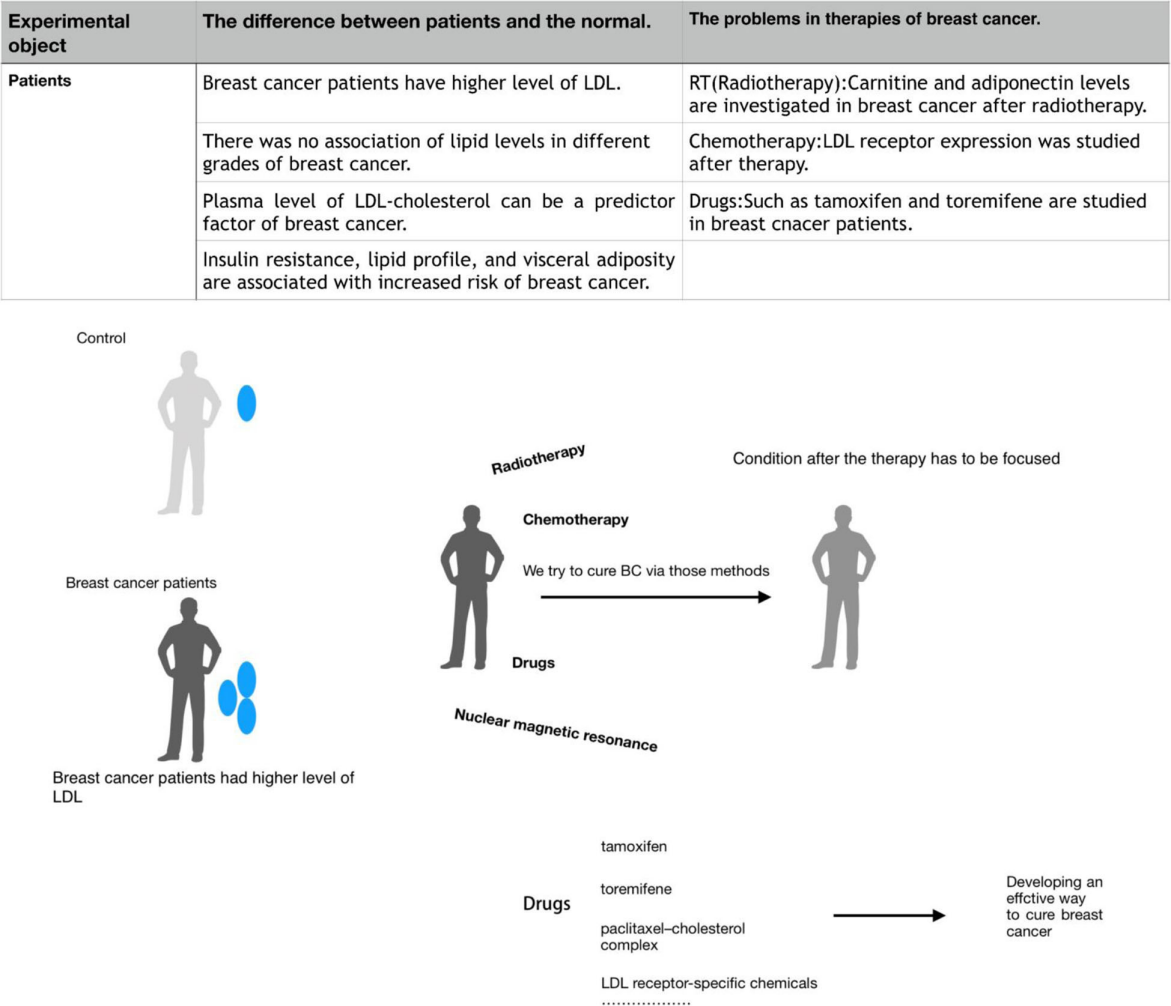
A lot of researches on breast cancer are made like differing the level between patient group and control group. There are serveral breast cancer therapy methods and more researches on after-therapy conditions need to be investigated

Gregory et al. investigated the function of statins on breast cancer. Among adherent statin users, mean LDL levels in subsequent years of follow up was significantly lower in comparison to the 1st year, while mean HDL levels were similar between observation periods [58–60]. Ji et al. evaluated the effect of atorvastatin on breast cancer. The two patient groups (placebo and atorvastatin) were well balanced, with no statistically significant differences in baseline characteristics. However, the statin group showed a significant decrease in cholesterol and LDL levels, suggesting medication involvement [61]. Although many preclinical and epidemiological results support the anticancer effects of statins, epidemiological evidence has not suggested an association between statin use and reduced incidence of breast cancer. Thomas et al. suggested that existing evidence was sufficient to perform a clinical trial on breast cancer using adjuvant therapy with statins, and they claimed that this trial should be performed as soon as possible [62]. The comparison of breast cancer between the patient group and the control group is indicated in Fig. 3.

### Summary

**Fig. 4:**
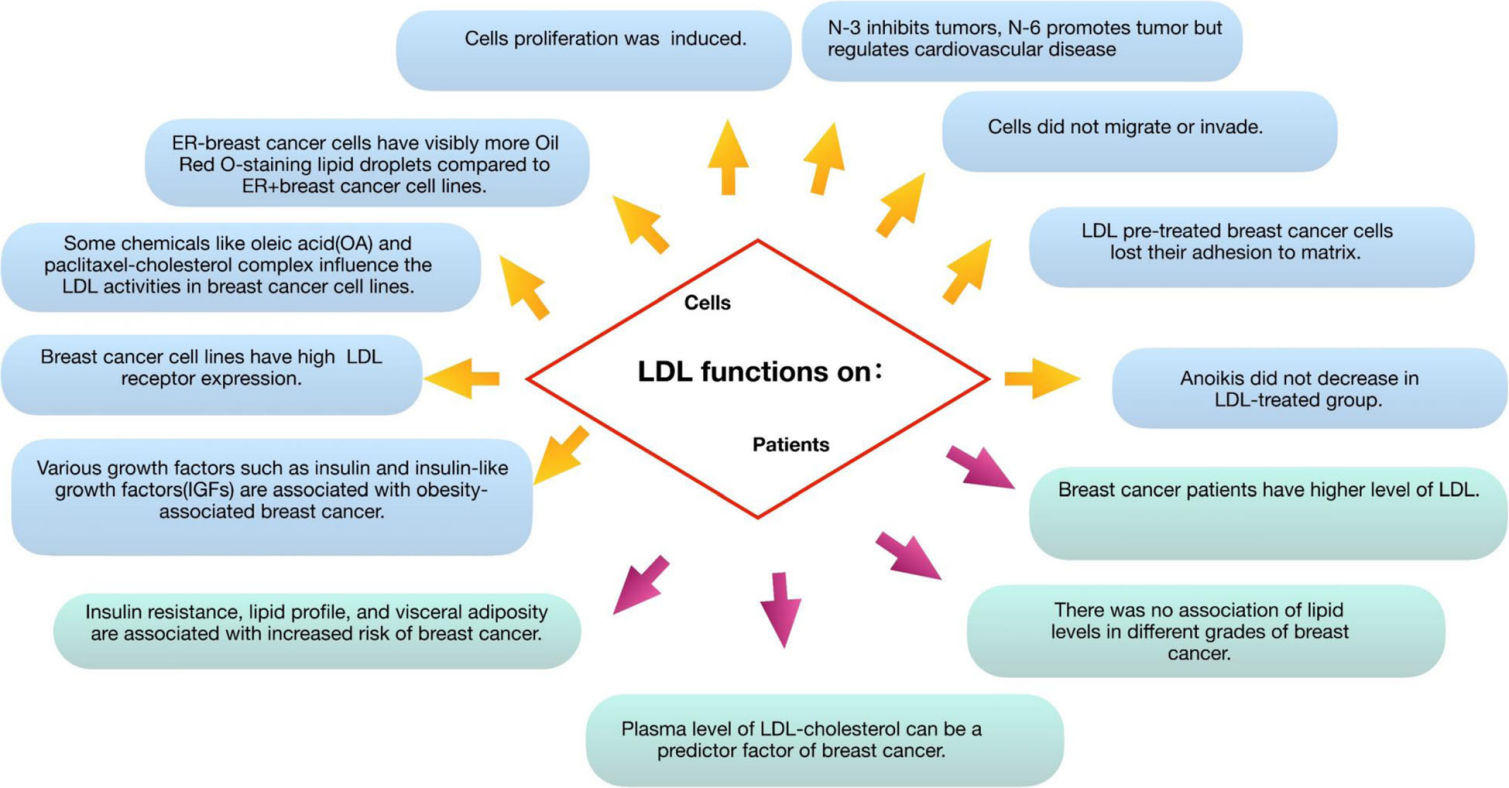
Summary on functions of LDL on both cells and patients

LDL is a lipoprotein particle that carries cholesterol from the liver to peripheral tissues and organs. LDL can be isolated from human peripheral blood by high-speed centrifugation. The treatment of MCF7, 4 T1, HS578T, MDA-MB-468, and MDA-MB-231 breast cancer cell lines with LDL resulted in increased proliferation and migration of certain types of breast cancer cells, and loss of adhesion. The influence on invasion was not remarkable, and anoikis of breast cancer cells was not decreased. Breast cancer patients have higher levels of LDL, which has become a predictor of breast cancer. Classical therapeutic approaches, such as radiotherapy and chemotherapy and their relationship with LDL were investigated resulting influenced and influencing the LDL level. Tamoxifen has an impact on the serum lipid profile including LDL in breast cancer patients, and toremifene is as effective and safe as tamoxifen for advanced breast cancer patients. The use of toremifene may be a great improvement in the process of developing new therapies. A growing body of evidence to support LDL will affect the development of breast cancer through certain signaling pathways and acting cytokines, which summarized in Table 1 and Fig. 4. Moreover, investigating LDL receptor-specific drugs may also provide a promising approach to improve the clinical therapy of breast cancer.

**Table 1:** Signaling pathways affected by LDL in the development of breast cancer

Pathway and Actived	Moleculars involved	Process regulated	Reference
PTEN-PIP3-AKT	ERK	Proliferation	[10, 19, 20]
	Akt	Proliferation, Metastasis	
DNAM1	CD226	Apoptosis	[19, 21, 22]
	Claudin7	Apoptosis, Metastasis	
	Ocludin	Metastasis	
	Integrinβ8	Invasion, Metastasis	
JNK	NMT1	Growth, Metastasis	[10, 63]
	TNF-alpha	Migration	
Wnt	LRP5/6	Proliferation	[36, 37, 64, 65]
	LRP1	Proliferation, Metastasis	
	SRFP	Proliferation	
	MMP9	Proliferation, Metastasis	
	DKK	Proliferation, Metastasis	
Y705	Stat3	Growth, Transformation	[32]
Rox	miR-21	Growth	[66]

## Data Availability

The datasets generated and/or analysed during the current study are available in the NCBI PubMed repository.

## Abbreviations

AKT: Protein Kinase B
ATP: Adenosine Triphosphate
CD xxx: Cluster Of Differentiation Family
ERK: Extracellular regulated protein kinases
HDL: High-density lipoprotein
JNK: c-Jun N-terminal Kinase
LDL: Low-density lipoprotein
OA: Oleic Acid
VLDL: Very Low-density lipoprotein
